# Pretreatment of Sulfonylureas Reducing Perihematomal Edema in Diabetic Patients With Basal Ganglia Hemorrhage: A Retrospective Case-Control Study

**DOI:** 10.3389/fneur.2021.736383

**Published:** 2021-10-22

**Authors:** Zhang Jingjing, Zhao Jingjing, Hui Bo, Wang Le, Wei Jingya, Wei Dong, Yang Fang, Jiang Wen

**Affiliations:** Department of Neurology, Xijing Hospital, Fourth Military Medical University (Air Force Medical University), Xi'an, China

**Keywords:** sulfonylureas, perihematomal edema, intracerebral hemorrhage, prognosis, case-control study

## Abstract

**Background:** The sulfonylurea receptor 1–transient receptor potential melastatin 4 (SUR1–TRPM4) channel is a target key mediator of brain edema. Sulfonylureas (SFUs) are blockers of the SUR1–TRPM4 channel. We made two assessments for the pretreatment of SFUs: (1) whether it associates with lower perihematomal edema (PHE) and (2) whether it associates with improved clinical outcomes in diabetic patients who have acute basal ganglia hemorrhage.

**Methods:** This retrospective case-control study was conducted in diabetic adults receiving regular SFUs before the onset of intracerebral hemorrhage (ICH). All of the patients received the clinical diagnosis of spontaneous basal ganglia hemorrhage. The diagnosis was confirmed by a CT scan within 7 days after hemorrhage. For each case, we selected two matched controls with basal ganglia hemorrhage based on admission time (≤5 years) and age differences (≤5 years), with the same gender and similar hematoma volume. The primary outcome was PHE volume, and the secondary outcomes were relative PHE (rPHE), functional independence according to modified Rankin Scale score and Barthel Index at discharge, and death rate in the hospital.

**Results:** A total of 27 patients (nine cases and 18 matched controls), admitted between January 1, 2009 and October 31, 2018, were included in our study. There was no significant association between SFU patients and non-SFU patients on PHE volumes [15.4 (7.4–50.2 ml) vs. 8.0 (3.1–22.1) ml, *p* = 0.100]. Compared to non-SFU patients, the SFU patients had significantly lower rPHE [0.8 (0.7–1.3) vs. 1.5 (1.2–1.9), *p* = 0.006]. After we adjusted the confounding factors, we found that sulfonylureas can significantly reduce both PHE volume (regression coefficient: −13.607, 95% CI: −26.185 to −1.029, *p* = 0.035) and rPHE (regression coefficient: −0.566, 95% CI: −0.971 to −0.161, *p* = 0.009). However, we found no significant improvement in clinical outcomes at discharge, in the event of pretreatment of SFUs before the onset of ICH, even after we adjusted the confounding factors.

**Conclusion:** For diabetic patients with acute basal ganglia hemorrhage, pretreatment of sulfonylureas may associate with lower PHE and relative PHE on admission. No significant effect was found on the clinical outcomes when the patients were discharged. Future studies are needed to assess the potential clinical benefits using sulfonylureas for ICH patients.

## Introduction

Intracerebral hemorrhage (ICH) accounts for 10% of all strokes. It is associated with high disability and mortality risks; therefore, it brings a huge burden to the society today (1). The physical effects of hematoma (mass effect) and secondary perihematomal edema (PHE) are major causes of severe neurological deficits and even death. Although symptomatic treatments, such as osmotic drugs, have been used on ICH patients, their therapeutic effects are insufficient. Therefore, the need for novel anti-edema drugs has become more urgent (2).

Among all mechanisms causing brain edema, blood-brain barrier hyperpermeability and cell swelling inducers have been the two main foci (2). Out of all the target mediators, a key potential candidate is the sulfonylurea receptor 1–transient receptor potential melastatin 4 (SUR1–TRPM4) channel. In response to the depletion of ATP by hypoxia, the SUR1–TRPM4 channel opens and releases an unregulated flow of ions and therefore causes brain edema (3). In rodent stroke models, sulfonylureas were confirmed as a blocker to the SUR1–TRPM4 channel. They reduce non-selective ion influx and excess brain water in peri-infarct regions (3). Recently, clinical trials suggested that, after an ischemic stroke, sulfonylureas reduce symptomatic hemorrhagic transformation (4, 5). Besides this, pretreated sulfonylureas were associated with smaller volumes of hematoma and PHE after ICH as well as improved discharge destination (6, 7).

Basal ganglia hemorrhage, the most common form of spontaneous ICH, is associated with a high mortality risk (8). In this study, we investigated the effects of sulfonylureas pretreatment on volumes of PHE on admission and clinical outcomes at discharge for diabetic patients with basal ganglia hemorrhage.

## Materials and Methods

### Patients and Data Collection

We conducted a retrospective case-control study of diabetic patients with acute ICH admitted at the Department of Neurology of the Xijing Hospital of Air Force Medical University, Xi'an, China, from January 1, 2009 to October 31, 2018. The patients included in the study were over 18 years old, had sulfonylureas before the onset, and were diagnosed as spontaneous basal ganglia hemorrhage as confirmed by computed tomography (CT) scan within the first 7 days. For each case, two matched controls with basal ganglia hemorrhage were selected based on the most recent admission data (≤5 years), with age difference within 5 years, the same gender, and similar hematoma volume. The patients were excluded if they had a modified Rankin Scale (mRS) score ≥3, they had a secondary ICH, or the cranial CT was unavailable upon admission. Midline shift was measured as the perpendicular distance between the septum pellucidum and the ideal midline (the line being coplanar with the falx cerebri) (9). The primary outcome was PHE volume, and the secondary outcomes were relative PHE (rPHE, a ratio of PHE to ICH volume), functional independence according to mRS score and Barthel Index at discharge, and death rate in the hospital. This study was approved by the Ethics Committee of Xijing Hospital (KY20182067-F-1). Given that this study was retrospective, informed consent was not available.

### Imaging Analysis

An independent neuroradiologist blindly measured the ICH and PHE volumes using the 3D slicer software (v.4.10.2; NIH US) based on the regions of interest from the head CT of the patients upon admission. The assessment of the evaluating agreement of the researchers was performed in another study from our group, and the results indicated a high agreement for our researchers (10). In addition, the volume of ICH and PHE on CT was measured using a semiautomatic volumetric algorithm in our study (11). rPHE was calculated using the following formula: rPHE = PHE volume/ICH volume.

### Statistical Analysis

The statistical analysis was completed using the Statistical Package for Social Science (version 19.0 for Windows; SPSS Inc., Chicago). Normally distributed data were described as mean (standard deviation) and analyzed by Student's t-test. Skewed data were described as median (interquartile range) and analyzed by Mann–Whitney U-test. Chi-square test or Fisher exact test was used for comparison between dichotomous data. Linear regression or binary logistic regression were performed for multivariate analysis. Statistical significance was set as a two-sided P < 0.05.

## Results

### Study Population

**Figure 1:**
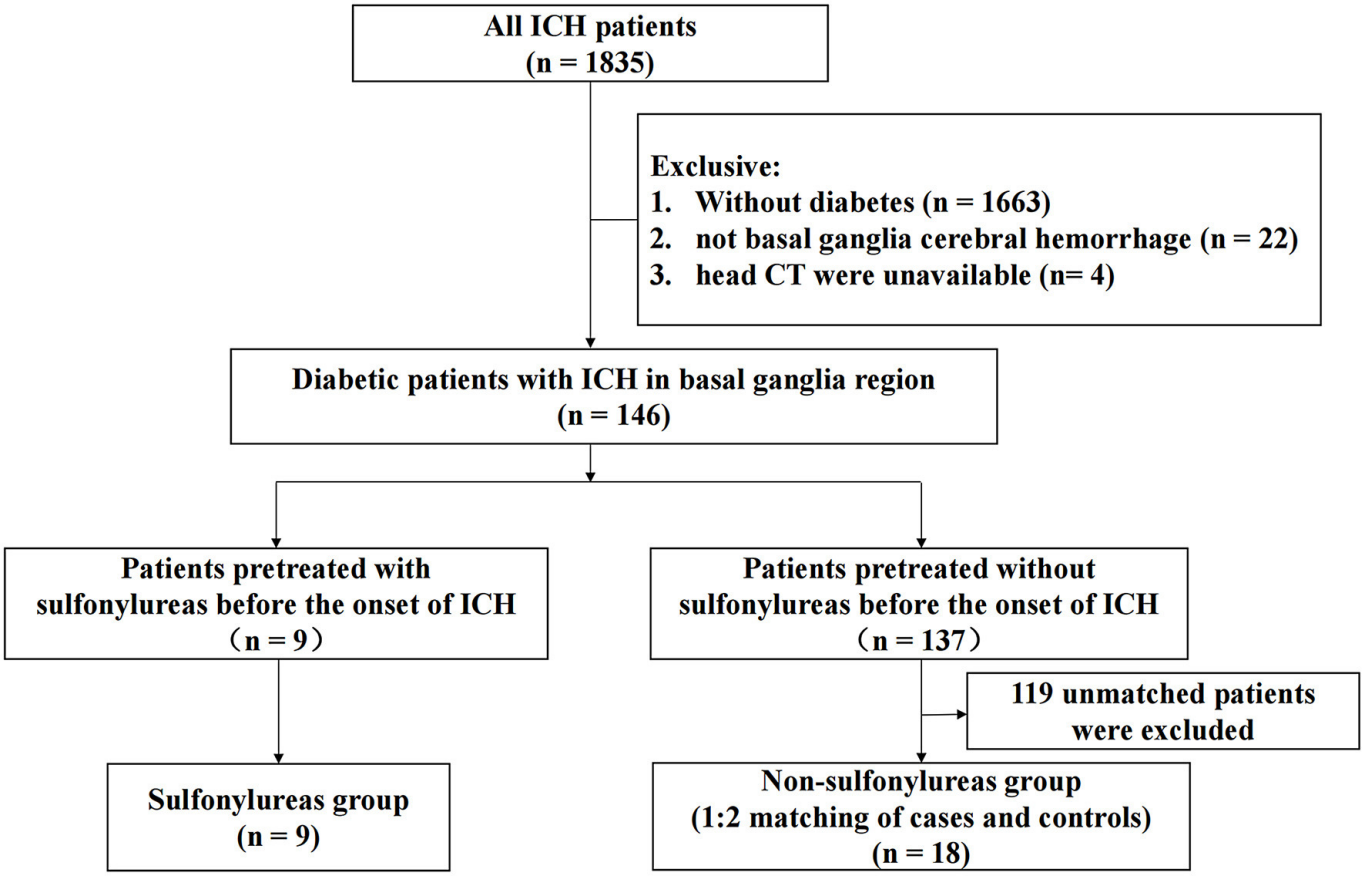
Flow diagram of the included and excluded patients.

A flow diagram of the included and excluded patients is provided in Figure 1. From January 1, 2009 to October 31, 2018, a total of 1,835 patients with spontaneous ICH were admitted to Xijing Hospital. Among them, 146 diabetic patients met the criteria of this study, which required ICH in the basal ganglia region. Nine patients pretreated with sulfonylureas before the onset of ICH were included in the sulfonylureas group (SFU patients), and 18 matched controls were selected (non-SFU patients). The average onset age of patients was 61 (51–71) years old. Out of all patients, 77.8% were male, and the average clinical scores were as follows: the NIHSS score was 13.0 (5.0–36.0), the Glasgow Coma Score (GCS) score was 14.0 (6.0–15.0), and the ICH score was 1.0 (0.0–3.0). The two groups did not differ significantly in baseline characteristics, such as age, gender, past history, medication history, blood pressure, clinical scores, and laboratory indicators, upon admission. The only major difference was that the time from onset to admission head CT in the sulfonylureas group was longer than that in the non-sulfonylureas group [1 (1–3) vs. 1 (1–1) days, *P* = 0.011] (Table 1). The clinical characteristics are shown in Table 1.

**Table 1:** Baseline characteristics of sulfonylureas (SFU) and non-SFU patients.

	**All patients**	**Non-SFU patients**	**SFU patients**	***P*-value**
	**(*N* = 27)**	**(*N* = 18)**	**(*N* = 9)**	
Age (years)	61 (51–71)	61 (51–68)	61 (45–72)	0.837
Male (*N*, %)	21 (77.8%)	14 (77.8%)	7 (77.8%)	1.000
Time from onset to admission head CT (day)^a^	1 (1–1)	1 (1–1)	1 (1–3)	0.011
ICH volume (ml)	11.4 (4.24–31.94)	12.7 (5.0–36.3)	11.4 (3.0–30.8)	0.440
**Past history**				
Stroke (*N*, %)	4 (14.8%)	4 (22.2%)	0 (0.0%)	0.268
Hypertension (*N*, %)	20 (74.1%)	13 (72.2%)	7 (77.8%)	1.000
Coronary heart disease (*N*, %)	3 (11.1%)	1 (5.6%)	2 (22.2%)	0.250
Hyperlipidemia (*N*, %)	2 (7.4%)	1 (5.6%)	1 (11.1%)	1.000
Alcoholism (*N*, %)	2 (7.4%)	1 (5.6%)	1 (11.1%)	1.000
Smoking (*N*, %)	6 (22.2%)	2 (11.1%)	4 (44.4%)	0.136
**Medication history**				
Antiplatelet agents (*N*, %)	3 (11.1%)	2 (11.1%)	1 (11.1%)	1.000
Anticoagulants (*N*, %)	1 (3.7%)	1 (5.6%)	0 (0.0%)	1.000
Statins (*N*, %)	3 (11.1%)	2 (11.1%)	1 (11.1%)	1.000
**Blood pressure on admission**				
SBP (mm Hg)^b^	160 (±27)	162 (±30)	156 (±23)	0.608
DBP (mm Hg)^b^	95 (±19)	96 (±20)	92 (±16)	0.635
MBP (mm Hg)^b^	117 (±20)	118 (±22)	113 (±17)	0.608
**Clinical scores on admission**				
NIHSS^a^	13.0 (5.0–36.0)	13.5 (5.0–34.0)	13.0 (3.0–36.5)	0.897
GCS^a^	14.0 (6.0–15.0)	14.0 (6.0–15.0)	15.0 (4.5–15.0)	0.956
ICH score^a^	1.0 (0.0–3.0)	1.0 (0.0–3.0)	1.0 (0.0–3.0)	0.978
**Laboratory indicators on admission**				
Blood glucose (mmol/L)^b^	12.2 (±5.3)	11.8 (±5.4)	13.1 (±5.4)	0.453
Hematocrit	0.4 (±0.45)	0.4 (±0.50)	0.4 (±0.37)	0.954
INR^b^	1.0 (±0.1)	1.0 (±0.1)	1.0 (±0.1)	0.905
Platelet (10^9^/L)^b^	176 (±56)	167 (±56)	193 (±56)	0.271
Serum creatinine (μmol/L)^b^	96.6 (±44.6)	105.1 (±52.2)	80.0 (±13.8)	0.161
Length of stay (day)^a^	13 (8–19)	13 (7–18)	13 (8–21)	0.938

*CT, computed tomography; SBP, systolic blood pressure; DBP, diastolic blood pressure; MBP, mean arterial pressure; NIHSS, National Institute of Health Stroke Scale; GCS, Glasgow Coma Score; ICH, intracerebral hemorrhage; INR, international normalized ratio*.

a *Median (quartile)*.

b *Mean ± standard deviation*.

### Treatment During Hospitalization

During hospitalization, more than half of the SFU patients continued to use sulfonylureas for blood glucose control, with a significantly higher rate than those in the non-SFU group (55.6 vs. 5.6%, *P* = 0.008). Between the two groups, there was no statistical difference in the treatment for blood pressure control nor in the use of osmotic drugs, hemostasis, craniotomy, drainage, mechanical ventilation, or other treatments.

### Imaging Characteristics

**Figure 2:**
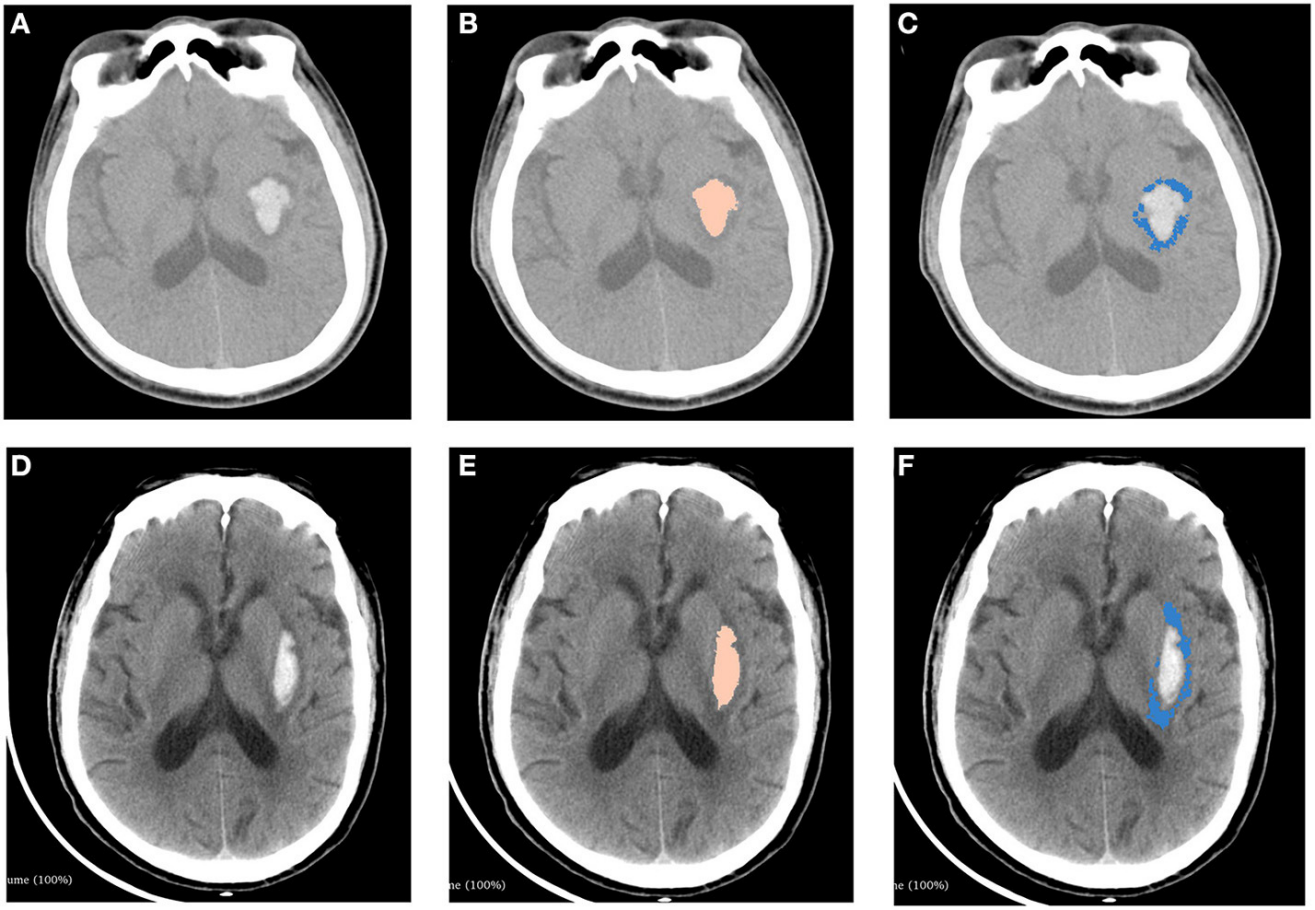
Measurements of hematoma and perihematomal edema using 3D slicer software package. Two patients with similar ICH volumes but different PHE volumes were shown in this figure. Top panel: A patient with ICH from non-SFU group. **(A)** original image of head CT. **(B)** hematoma (5.0 ml) marked with pink. **(C)** PHE (6.7 ml) marked with blue. Bottom panel: A patient with ICH from SFU group. **(D)** original image of head CT. **(E)** hematoma(4.9ml) marked with pink. **(F)** PHE (4.2 ml) marked with blue. ICH, intracerebral hemorrhage; PHE, perihematomal edema.

The measurements of hematoma and PHE using 3D slicer software package is provided in Figure 2. We found that there was no significant association between SFU patients and non-SFU patients on PHE volume [15.4 (7.4–50.2) ml vs. 8.0 (3.1–22.1) ml, *p* = 0.100]. However, the rPHE in SFU patients was significantly lower [0.8 (0.7–1.3) vs. 1.5 (1.2–1.9), *p* = 0.006], while there was no significant difference between the two groups in hematoma breaking into ventricles and midline shift. Table 2 shows the results of the first head CT upon admission of the SFU and non-SFU patients.

**Table 2:** Imaging characteristics on admission of sulfonylureas (SFU) and non-SFU patients.

	**Non-SFU patients**	**SFU patients**	***P*-value**
	**(*N* = 18)**	**(*N* = 9)**	
PHE volume (ml)^a^	15.4 (7.4–50.2)	8.0 (3.1–22.1)	0.100
rPHE^a^	1.5 (1.2–1.9)	0.8 (0.7–1.3)	0.006
Hematoma breaking into ventricles (*N*, %)	6 (33.3%)	2 (22.2%)	0.676
Midline shift (*N*, %)	7 (38.9%)	3 (33.3%)	1.000

a *Median (quartile)*.

In order to clarify the influence of confounding factors on our results, we carried out collinearity statistics in regression analysis. The results showed that there was no multicollinearity in the regression analysis in our study (Table 3). After adjusting for confounding factors, we found that sulfonylureas significantly associated with a lower PHE volume (regression coefficient: −13.607, 95% CI: −26.185 to −1.029, *p* = 0.035) and rPHE (regression coefficient: −0.566, 95% CI: −0.971 to −0.161, *p* = 0.009) (Table 4).

**Table 3:** Collinearity statistics of confounding factors in regression analysis.

	**PHE**		**rPHE**	
	**Tolerance**	**VIF**	**Tolerance**	**VIF**
Age	0.654	1.530	0.654	1.530
Gender	0.536	1.864	0.536	1.864
Time from onset to admission head CT	0.676	1.480	0.676	1.480
ICH volume	0.276	3.619	0.276	3.619
Hematoma breaking into ventricles	0.626	1.598	0.626	1.598
Midline shift	0.213	4.701	0.213	4.701

*VIF, variance inflation factor*.

*The collinearity in regression analysis can be judged by two indicators: (1) when VIF ≥ 10, it indicates that there is serious multicollinearity between independent variables; and (2) when tolerance is <0.1, collinearity may be serious*.

**Table 4:** Analysis of the associations between pretreatment of sulfonylureas use, perihematomal edema (PHE) volume and rPHE in sulfonylureas (SFU) and non-SFU patients.

	**Regression coefficient (95% CI)**	***P-value***	**Adjusted regression coefficient (95% CI)**	**Adjusted *P*-value**
PHE volume	−17.816 (−36.874 to 1.243)	0.066	−13.607 (−26.185 to −1.029)	0.035
rPHE	−0.593 (−0.977 to −0.210)	0.004	−0.566 (−0.971 to −0.161)	0.009

*Adjusted P-value by multiple linear regression analysis for confounders, including age, gender, time from onset to admission head CT, ICH volume, hematoma breaking into ventricles, and midline shift*.

### Clinical Outcomes

The average mRS score was 4 at the time the patients were discharged in both groups; no significant differences were found (*P* = 0.831). In neither group did we find any significant association between pretreatment of sulfonylureas and in-hospital mortality, the number of patients with mRS ≥ 3, or Barthel Index at discharge. This remained true even after we adjusted for confounding factors (age, gender, NIHSS and GCS on admission, imaging data including ICH volume, PHE volume, hematoma breaking into ventricles and midline shift, and sulfonylureas during hospitalization).

## Discussion

The retrospective case-control study showed that pretreatment of sulfonylureas significantly associated with lower PHE and rPHE in diabetic patients who have acute basal ganglia hemorrhage. However, there was no significant difference on clinical outcomes between SFU and non-SFU patients at discharge.

Basal ganglia hemorrhage accounts for 50–70% of spontaneous intracerebral hemorrhage, and 35–44% of basal ganglia hemorrhage cases are due to hypertension (12, 13). In our study, we did not find any significant differences in patients with hypertension between the SFU and non-SFU groups. Regarding the use of sulfonylureas during hospitalization, significantly more patients in the SFU group received it than in the non-SFU group. Moreover, we found no difference in other treatments between the two groups.

Our findings provided evidence that an association existed between the pretreatment of sulfonylureas prior to the onset of ICH and decreased PHE and rPHE in the acute stage. This is in accordance with a previous clinical trial (7). Preclinical studies proved that sulfonylureas blocked the SUR1–TRPM4 channel from allowing ions to enter into cells. This resulted in reducing the formation of cytotoxic edema and vasogenic edema; then, sulfonylureas inhibited the secretion of the inflammatory mediator MMP-9 and eventually benefit ICH (14, 15). After adjusting for confounders, our results showed that the PHE volume and rPHE in SFU patients were smaller than those in non-SFU patients. In addition, in order to more accurately describe the effect of sulfonylureas on PHE, we used ICH volume as one of the matching factors instead of an outcome indicator.

For patients pretreated with sulfonylureas before admission, one previous study described improved discharge destination, and another study found that pretreatment with sulfonylureas was significantly associated with a lower likelihood of unfavorable functional outcome (mRS ≥ 3) at discharge (6, 7). Similar to a previous study (6), we used both mRS score and Barthel Index at discharge as parameters to assess the functional outcomes. However, pretreatment of sulfonylureas before the onset of ICH showed no significant improvement in clinical outcomes at discharge, even after we had adjusted for the confounding factors. One reason for the difference between our results and the two studies mentioned above may be that we limited the location of ICH to the basal ganglia; in those studies, the site of hematoma was not clearly defined. However, it was a determinant of functional outcomes (16).

Several potential limitations need to be taken into account. Firstly, our study was designed as a retrospective study with a relatively small sample size; however, with our strict inclusion criteria as well as the 1:2 matching of cases and controls, it could represent the broader populations of ICH patients with diabetes. Secondly, due to the widespread use of different oral antidiabetic medications, the rate of sulfonylureas administration has largely decreased in Chinese diabetic patients, especially in the urban areas. For this reason, we studied all the sulfonylureas without limiting to a single drug. Thirdly, since it was difficult to obtain long-term follow-up data, the primary outcome of our study was based on imaging data rather than clinical outcomes. Lastly, we only analyzed admission CT scans because of a range of available antidiabetic agents during hospitalization. That might have different effects on outcomes, but it was what we had to do in real-world medical care. The limitations mentioned above must be recognized, but we believe that they would not invalidate the overall findings.

In conclusion, our study suggested that, for diabetic patients with acute basal ganglia hemorrhage, pretreatment of sulfonylureas may associate with lower PHE and relative PHE on admission. However, it has no significant effect on clinical outcomes at discharge. Future studies are needed to assess the potential clinical benefits in using sulfonylureas for ICH patients.

## Data Availability Statement

The raw data supporting the conclusions of this article will be made available by the authors, without undue reservation.

## Ethics Statement

The studies involving human participants were reviewed and approved by the Ethics Committee of Xijing Hospital. Written informed consent for participation was not required for this study in accordance with the national legislation and the institutional requirements.

## Author Contributions

This trial was conceived by YF and JW. ZhanJ and ZhaoJ collected the data, performed statistical analysis, and wrote the first version of the manuscript, with the advice from YF and JW. HB, WL, WJ, and WD assisted in data collection, statistical analysis, and manuscript modification. All authors are responsible for the designation and conduction of this study as well as the data analysis, the drafting, and the reviewing of the final content of the paper.

## Conflict of Interest

The authors declare that the research was conducted in the absence of any commercial or financial relationships that could be construed as a potential conflict of interest.

## Publisher's Note

All claims expressed in this article are solely those of the authors and do not necessarily represent those of their affiliated organizations, or those of the publisher, the editors and the reviewers. Any product that may be evaluated in this article, or claim that may be made by its manufacturer, is not guaranteed or endorsed by the publisher.
